# Toxicity Testing in the 21^st^ Century: Defining New Risk Assessment Approaches Based on Perturbation of Intracellular Toxicity Pathways

**DOI:** 10.1371/journal.pone.0020887

**Published:** 2011-06-20

**Authors:** Sudin Bhattacharya, Qiang Zhang, Paul L. Carmichael, Kim Boekelheide, Melvin E. Andersen

**Affiliations:** 1 Program in Chemical Safety Sciences, The Hamner Institutes for Health Sciences, Research Triangle Park, North Carolina, United States of America; 2 Safety and Environmental Assurance Centre, Unilever, Colworth Science Park, Sharnbrook, Bedford, United Kingdom; 3 Department of Pathology and Laboratory Medicine, Brown University, Providence, Rhode Island, United States of America; East Carolina University, United States of America

## Abstract

The approaches to quantitatively assessing the health risks of chemical exposure have not changed appreciably in the past 50 to 80 years, the focus remaining on high-dose studies that measure adverse outcomes in homogeneous animal populations. This expensive, low-throughput approach relies on conservative extrapolations to relate animal studies to much lower-dose human exposures and is of questionable relevance to predicting risks to humans at their typical low exposures. It makes little use of a mechanistic understanding of the mode of action by which chemicals perturb biological processes in human cells and tissues. An alternative vision, proposed by the U.S. National Research Council (NRC) report *Toxicity Testing in the 21^st^ Century: A Vision and a Strategy*, called for moving away from traditional high-dose animal studies to an approach based on perturbation of cellular responses using well-designed *in vitro* assays. Central to this vision are (a) “toxicity pathways” (the innate cellular pathways that may be perturbed by chemicals) and (b) the determination of chemical concentration ranges where those perturbations are likely to be excessive, thereby leading to adverse health effects if present for a prolonged duration in an intact organism. In this paper we briefly review the original NRC report and responses to that report over the past 3 years, and discuss how the change in testing might be achieved in the U.S. and in the European Union (EU). EU initiatives in developing alternatives to animal testing of cosmetic ingredients have run very much in parallel with the NRC report. Moving from current practice to the NRC vision would require using prototype toxicity pathways to develop case studies showing the new vision in action. In this vein, we also discuss how the proposed strategy for toxicity testing might be applied to the toxicity pathways associated with DNA damage and repair.

## Introduction

The goal of toxicity testing should be the collection of appropriate results from test systems in order to assess the likely risks posed to human populations at ambient exposure levels; i.e., provide the data inputs necessary for a realistic assessment of human risk. Traditionally, these objectives were met by high-dose testing in experimental animals with specific approaches for extrapolation from high to lower doses and from the experimental animals to the human population. Modern toxicology has incorporated techniques emerging from the field of molecular biology in the 1980s and 1990s for assessment of modes of action and target identification. However, the gold standard against which toxicity testing methods are evaluated has remained largely unchanged over the past five decades: organism-level responses (e.g., hepatotoxicity, cancer, reproductive/developmental toxicity, and neurotoxicity) deemed to serve as measures of adverse responses in high-dose studies with homogeneous groups of laboratory animals.

In the U.S., extrapolation from these high-dose animal toxicity tests to expected responses in humans from low-dose chemical exposure is based on a variety of uncertainty factors or on linear extrapolations through a zero dose. Not surprisingly, there has been broad disaffection with this approach from both regulatory agencies and regulated communities. These traditional methods are expensive, exorbitant in their use of animals, and have low throughput. Although over the past 30 years there have been attempts to incorporate pharmacokinetics and modes of action for certain high-value or high-liability compounds into the risk assessment process, these research efforts have been costly and have had limited success in changing the existing regulatory framework. In addition, most of these research efforts have focused more on explaining high-dose rodent effects than on understanding the biological basis for dose-response relationships expected in humans exposed to chemicals at relevant environmental levels.

In response to these concerns, in 2004 the U.S. Environmental Protection Agency (U.S. EPA) and the National Toxicology Program of the U.S. National Institute of Environmental Health Sciences commissioned a committee of the National Academy of Sciences to evaluate current toxicity testing methods and recommend changes that would take into account the new understanding of human biology and emerging testing technologies. The culmination of the work of the committee on toxicity testing for environmental agents was a report issued by the U.S. National Research Council (NRC) in 2007: *Toxicity Testing in the 21^st^ Century: A Vision and a Strategy* (TT21C) [1]. At the center of the vision for transforming toxicity testing described in this report is a reorientation of such testing to evaluating the responses of toxicity pathways, i.e., normal cellular signaling pathways that can be perturbed by chemical exposures, in well-designed *in vitro* assays using human cells. This new paradigm would replace the current approach of relying almost exclusively on high-dose phenotypic responses in animals.

**Figure 1:**
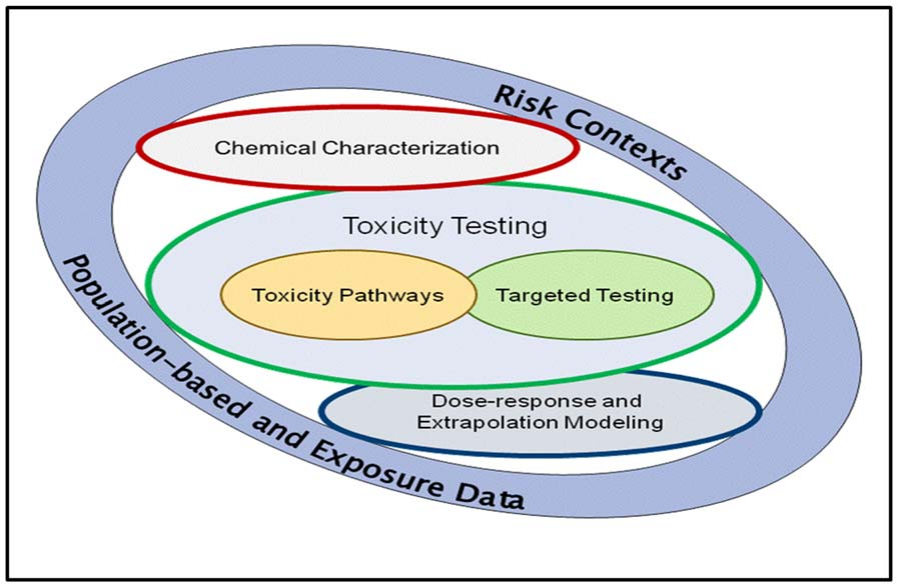
Schematic of components required to implement the new vision of toxicity testing in the 21^st^ century (*needs permission from NRC TT21C report*). The core components of the TT21C vision relate to testing for biological activity of compounds in toxicity pathway assays and to dose-response and extrapolation modeling using computational systems biology and pharmacokinetic tools. In the period of transition from current practice to the new vision some targeted testing in animals might be required. Targeted testing might also allow evaluation of target pathways and identification of metabolites. Assessing likely metabolism remains a challenge for the full implementation of the TT21C vision.

The new approaches based on evaluation of *in vitro* assays that cover a range of toxicity pathways would enable testing across large concentration ranges for multiple modes of action and provide sufficient sensitivity for detection of biological effects at low concentrations unobtainable with conventional animal studies. The schematic of the components involved in this vision (Figure 1) includes toxicity pathway testing, limited targeted testing in animals required until a comprehensive suite of pathway tests is completed and validated, and dose-response and extrapolation modeling for interpreting the *in vitro* assay results for assessment of risk to human health. That the committee was not tasked to develop alternatives to animal testing is a point deserving of some emphasis. Rather, it was directed to design an appropriate, modern approach to toxicity testing. In the final analysis, the committee argued that *in vitro* assays using human cells or tissue surrogates would be much preferred to conventional studies with animals; nonetheless, one of the testing design criteria stressed in its report was to use the fewest animals necessary in the most humane manner if targeted *in vivo* testing was pursued.

### New directions in toxicity testing from the European Union

During the time frame that the National Academy of Sciences committee was in deliberation, regulatory changes in the European Union (EU) were moving the testing of cosmetics ingredients toward the elimination of animal testing altogether. Notably, the European 7^th^ Amendment to the Cosmetics Directive (passed in 2003) has restricted the use of animal tests for a variety of end points [2]. An initial ban on acute toxicity testing in animals, including *in vivo* genotoxicity testing, came into effect in 2009. By 2013 the legislation will dictate that no animal tests (including repeat-dose studies, reproductive toxicity testing, and carcinogenicity bioassays) can be conducted on cosmetic ingredients intended for the European market.

Elsewhere, breakthrough thinking on how to address this issue paralleled the NRC report. Fentem and colleagues, for example, have highlighted the need for a paradigm shift in toxicology to risk assessments that support decisions about consumer safety without the need to generate data through animal tests [3]. Their approach was based on the concept that information derived from existing and new non-animal models can be interpreted in terms of potential for human pathology, following realistic exposure levels, rather than replacement of animal tests on a like-for-like basis.

### Problems faced by EU cosmetics manufacturers in assessing genotoxicity

As a result of the European 7^th^ Amendment (2003) [2] there is now a ban in the EU on animal testing for the assessment of genotoxic effects of chemical ingredients intended for cosmetic products. In practical terms, this has meant the cessation of several tests, including the widely used bone marrow micronucleus assay in rodents [4]. For now, however, the possibility remains for incorporating the micronucleus end point into repeat-dose general (systemic) toxicology assessments in rodents along with other genetic toxicology end points such as the Comet assay, but only until 2013 [5]. Although the 2-year rodent cancer bioassay is very rarely used in the testing of cosmetic ingredients, this is also included in the 2013 ban. These changes leave the cosmetics industry with an innovation-constrained and uncompetitive dilemma. First, *in vitro*-only genetic toxicology assay strategies have a high irrelevant-positive rate [6], [7], and many common biochemical products (including those derived from food, e.g., flavonoids) [8], [9], would be erroneously rejected for commerce if *in vitro* regulatory tests alone were used. The high false-positive rate occurs because of the inherent nature of the current assay. Since these assays are used purely in a hazard identification mode, the label of “genotoxicity,” applied to a chemical by the tests irrespective of dose, would lead to the rejection of that chemical if no follow-up testing is conducted.

To see *in vitro* genetic toxicology tests as the problem, however, is to miss the important point that they are in fact one of the most refined sets of tools available to toxicologists to define the human pathology concern (cancer) in terms of the toxicity pathways that may precede it; i.e., mutagenicity, clastogenicity, and aneugenicity. It is only the lack of a dose-response element in the use of genetic toxicology tests that limits their current use in risk assessment. This discrepancy should be the focus of research in this area from the TT21C perspective in order to overcome the obstacles of extrapolating from *in vitro* to *in vivo*. A problem in this respect is the current underutilization of information on the biological mechanisms for genotoxicity and on the homeostatic processes that attenuate the effects at low doses in the whole organism. Only by recognizing the current limitations in the understanding of these processes and by embracing computational modeling that mechanistically addresses the effects of low-dose perturbations will we be able to move forward.

The disciplines of genetic toxicology and assessment of risk for carcinogenicity are clearly prime candidates for change in line with the TT21C vision. Genetic toxicology has long involved the use of *in vitro* assays for primary assessment, but in keeping with the ban on *in vivo* follow-up tests on cosmetic ingredients the model for the future is an exclusive reliance on *in vitro* tests without confirmatory animal studies for carcinogenicity or *in vivo* mutations. Hence, the drive to alternatives in the EU and in the US vision of TT21C, while developed for different requirements – sparing animals versus redesign of test methods – are following very similar trajectories and have reached a similar position with respect to the changes needed in toxicology.

### US activities subsequent to publication of the NRC report

In 2008, the U.S. EPA Office of Research and Development, the National Toxicology Program at the U.S. National Institute of Environmental Health Sciences, and the U.S. National Chemical Genomics Center initiated a partnership to advance toxicity testing towards the goals espoused in the NRC report [10]. A “Future of Toxicity Testing Workgroup” was announced in a 2008 paper in *Science* to design a research strategy that would move the science and technology used in toxicity testing away from reliance on high-dose animal testing towards high-throughput *in vitro* assays designed to detect perturbations in toxicity pathways [10]. The paper stressed that high-throughput *in vitro* screening assays could be used for (a) prioritizing chemicals for *in vivo* testing, (b) predicting the results of animal studies, and (c) assisting in risk assessment. While this interagency initiative calls for relatively modest changes compared to the full TT21C vision of having appropriately designed *in vitro* assays as the core element of risk assessment, many of the steps outlined for assay design, technology implementation, and database development are essential for any program that aims to replace animal testing with suites of *in vitro* assays.

In another development, the U.S. EPA has published *The U.S. Environmental Protection Agency's Strategic Plan for Evaluating the Toxicity of Chemicals* (http://www.epa.gov/spc/toxicitytesting/). This report calls for a stepwise approach for developing *in vitro* testing for chemical screening as well as the development of “virtual tissue” computational models for predicting toxicity, leading eventually to using their results for various risk management decisions [11].

### Broader interest in the TT21C vision

Two of the National Academy of Sciences committee members recently described the key attributes of the NRC report as part of a commentary in *Toxicological Sciences* [12]. In addition, the journal's editors provided an overview of the NRC report [13] and invited seven commentaries from researchers in various sectors of the toxicology community [14], [15], [16], [17], [18], [19], [20]. These articles appeared in the journal throughout 2009 and in early 2010, and discussed the benefits and shortcomings of the new scientific methods outlined in the NRC report for toxicity testing and risk assessment. The commentaries expressed different degrees of both support and skepticism for the TT21C vision, and described many of the scientific challenges and changes needed to make the vision a reality. The two authors from the National Academy of Sciences committee had an opportunity to respond to these varied perspectives [21].

**Figure 2:**
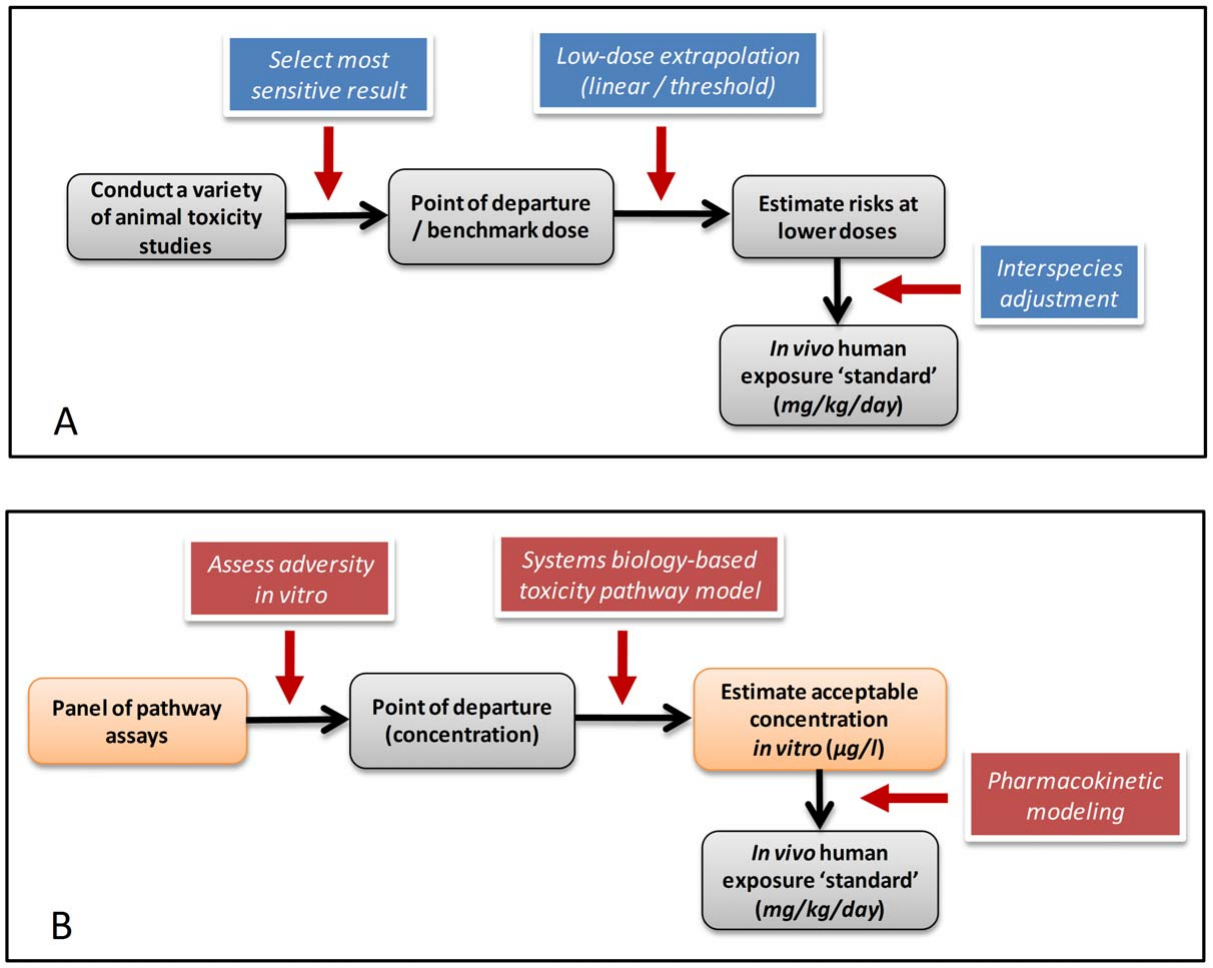
Comparison of current (A) and proposed (B) toxicity testing paradigms. The current approach (Panel A) to setting regulatory standards involves interpretation of the most sensitive end point observed in animal studies. Low-dose extrapolation requires obtaining a point of departure from the results of the animal studies and the use of either linear or threshold extrapolation plus application of uncertainty factors to the point of departure. Other extrapolations between species or across exposure routes are sometime conducted with pharmacokinetic modeling of the tissue doses that are associated with adverse effects. A similar sequence of steps can be envisioned for setting standards based on pathway assays (Panel B). Likely hazards are determined by the sensitivity of the various toxicity pathway assays; extrapolations require assessing adverse consequences of *in vitro* exposures and use of computational systems biology pathway (CSBP) and pharmacokinetic modeling to set a standard for human exposure related to mg/kg/day ingested or ppm (parts per million) in the inhaled air.

Some overarching issues emerged from the commentaries: (a) Because not all responses observed in *in vitro* assays will be adverse, how will a determination be made as to which of the responses obtained warrant attention from a risk assessment perspective? (b) Because *in vivo* responses frequently require multi-tissue interactions that will be absent from *in vitro* testing assays, how can apical responses in intact mammalian systems be predicted on the basis of *in vitro* data? (c) Given that the ultimate goal of characterizing risk is the establishment of a recommended guideline for human exposure, traditionally done by extrapolation of animal toxicity data to humans (see Figure 2A), why was the NRC report silent on the manner in which *in vitro* results would be used for deriving guidelines for human exposure based on perturbations of toxicity pathways? (d) Finally, how can such fundamental, pervasive changes in the way we test for toxicity (Figure 2B) be achieved in a smooth, efficient manner? Are such changes even possible given the rather hidebound regulatory environment? In the present article we comment on each of these issues in the context of developing an example of a human risk/safety assessment based on examination of a toxicity pathway associated with DNA damage.

## Results and Discussion

### Examining a prototype pathway: DNA-damage repair

The gold standard for predicting carcinogenicity is the 2-year rodent bioassay. Although it has evolved somewhat over time, this test has become standardized by the U.S. National Toxicology Program. In its standard protocol, this program conducts 2-year bioassays in two species (rats and mice), beginning treatments at 5–6 weeks of age in both sexes with three doses and a control. The 2-year bioassay is supported by preliminary shorter-term animal studies that evaluate data on absorption, distribution, metabolism, and excretion that are important to setting the dose. The highest dose tested is a maximum tolerated dose (MTD), with half or one quarter of the MTD usually selected as additional doses. The MTD is estimated from the short-term studies and is operationally defined as the highest dose that does not significantly increase lethality or induce more than a 10% weight loss when compared to controls.

The use of the MTD is usually justified by the argument that high doses increase the likelihood of seeing a response given the group sizes used, i.e., about 100. Unfortunately, the MTD frequently induces biological responses (such as cytotoxicity) that will not occur at lower doses in animals or humans. The induction of irrelevant responses in rodents exposed at high doses creates a significant problem of interpretation, i.e., separating those effects that are due only to high doses from those that would persist at lower levels of exposure. This interpretive problem is made more difficult by the lack of human data for almost all of the identified rodent carcinogens.

### Cancer modes of action

Carcinogenicity is believed to result from a progressive accumulation of mutations that select for a growth/survival advantage in affected cells. In defining *in vitro* tests for detecting carcinogens the goal has been to identify and quantify alterations in the molecular pathways associated with the generation and fixation of mutations. The two major categories of chemical carcinogens are *DNA-reactive carcinogens* (genotoxic carcinogens) and *non-genotoxic carcinogens* that alter DNA indirectly through other effects on the cell [22]. Genotoxic carcinogens, or their metabolites, are usually electrophiles that can directly react with DNA to form adducts, and thus they can directly initiate the neoplastic transformation of a normal cell. On the other hand, non-genotoxic carcinogens can act through pathways associated with the modulation of reactive oxygen species, proliferation, apoptosis, endocrine controls, or immune surveillance to promote pre-initiated tumor formation. Risk assessment procedures treat genotoxic carcinogens as having no threshold, while non-genotoxic carcinogens are considered to have a threshold, leading to regulation of these chemicals through the use of safety factors. This distinction between genotoxic and non-genotoxic carcinogens is important in establishing exposure guidelines for chemicals.

Unfortunately, the bulk of the immense body of mechanistic work conducted to study carcinogenic responses for risk assessment has been restricted to the manner of extrapolating to lower doses (linear versus threshold) or to determining that certain rodent cancers should not be regarded as relevant for humans (such as hydrocarbon nephropathy in male rats, or thyroid tumors in rats associated with enhanced clearance of thyroid hormone). The challenge faced with mode-of-action studies *in vivo* or with the proposed *in vitro* toxicity pathway assays is to bring a *fundamentally new perspective to dose-response extrapolation* rather than to simply choose between linear low-dose and threshold approaches. Ideally, the new assays for toxicity testing and for test interpretation should provide information to allow mechanistic prediction of the shape of the dose-response curve over broad ranges of concentration, thereby enabling assessment of a point of departure for risk assessment.

### Approaches involving toxicity pathways applied to DNA-reactive compounds

The goal of toxicity testing should not be to predict high-dose outcomes in rodents but rather the more accurate prediction of the likely risks of low-dose exposures in human populations. With respect to mechanistic dose-response behaviors for DNA-reactive compounds, it would be necessary to assess the conditions under which perturbations of DNA structure are expected to propagate into fixed mutations in the genome and provide altered cells with the potential for autonomous growth. Identifying this transition from the condition where DNA-repair processes can control any induced damage to one where there are sufficiently large degrees of damage leading to mutations [23] would be a key step in assessing “*adversity*” at the cellular level for DNA-reactive compounds.

Consistent with the procedures presented in the NRC report, the risk/safety assessment of chemicals with potential impact on human health, e.g. ingredients in cosmetics, should start with an initial assessment of the nature of the chemical and the expected levels of human exposure. All available information needs to be taken into account, such as read-across to similar molecules, QSAR (quantitative structure-activity relationship) alerts, and any other available data. If genotoxicity testing is required, a test-battery covering mutation, clastogenicity and aneuploidy would be adequate as the initial part of the test protocols for *in vitro* toxicity pathways. A broader suite of pathway assays would provide a catalogue of likely modes of action and of the relative ability of compounds to activate particular pathways based on their potency (by identifying an effective concentration causing 50% or 10% maximum responses, i.e., an EC50 or EC10). Such screening over multiple molecular targets is common with pharmaceutical compounds and has been pursued by the U.S. EPA in its ToxCast program [24]. Comparison of EC50s or EC10s across a suite of inclusive pathway assays will allow for identification of primary mode(s) of action for low-dose exposures. For instance, compounds that are found to have lower EC50s in DNA–reactivity-oriented assays than in assays for other toxicity pathways would be considered likely to have mutagenic and carcinogenic potential as the primary mode of action in humans with low-dose exposures. For cases where higher EC50s are found for mutagenicity than for other pathway activation, the conclusion would be that mutagenicity is likely to be a secondary effect, with primary modes of action suggested by more sensitive pathways such as oxidative stress or activation of receptor-mediated pathways.

However, this enumeration of activities across a suite of tests, and the doses at which they produce perturbations of pathways, would not by itself define ‘adversity’ at a cellular or molecular level; instead it would only define likely hazards. This consideration of all pathway assay EC50s would provide a more qualitative assessment of likely apical outcomes and a relative measure of *in vitro* potency, but conclusions about adversity will require a more quantitative integration of the results of pathway assays examining different aspects of DNA–damage-and-repair pathway function.

### Degrees of perturbations as a measure of adversity *in vitro*

**Figure 3:**
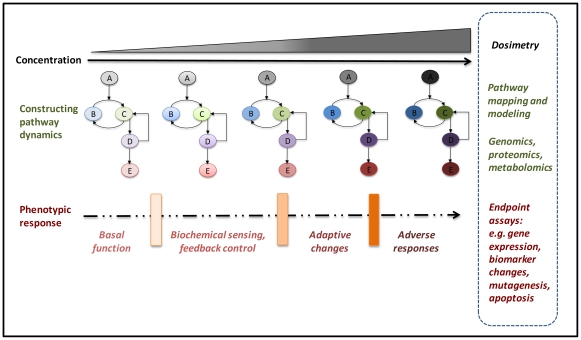
A schematic figure illustrating progressive activation of a prototype toxicity pathway, with attendant discrete phenotypic transitions. Text on right of figure shows proposed approaches for characterizing discrete transitions through multiple cellular phenotypes, i.e., (1) basal function, (2) minimally perturbed cellular states, (3) upregulation of adaptive, homeostatic gene batteries, and finally (4) overtly adverse states with excessive pathway perturbations. The structure of the circuitry with various embedded, nonlinear feedback loops is schematized in the middle panels. In the context of *in vitro* assay design, varying free concentrations of a test compound in the test media lead to increasing activation of pathway component (signaling protein) A, thereby driving pathway perturbations of signaling components B and C, and further signaling events downstream of C. Each of these steps is expected to be associated with specific alterations in gene expression, phenotypic read-out and pathway activation, identified by CSBP modeling. As the concentration increases, various portions of the network would be sequentially activated until full activation was achieved (indicated by progressively darker shading of pathway components). Full pathway activation triggers a robust response throughout the pathway circuitry, leading to adverse outcomes measurable in the cellular assay.

Toxicology has long made use of *in vitro* test batteries to predict specific toxicity to target organs. The goal of mode-of-action, human biology-based testing should not be to generate batteries of tests to provide a prediction of test results on animal toxicity for various end points. Instead, these methods should be intended to determine regions of exposure that will not cause any adverse responses in exposed *human* populations. These panels of assays, evaluating specific pathway targets such as DNA damage and repair, would be designed to capture increasing degrees of perturbation (Figure 3). The scheme shown in Figure 3 would require several types of assays for increasing severity or a single assay that could evaluate different regions of response. Each test in the panel would be designed to provide dose-response over broad ranges of treatment and assess some aspect of pathway perturbation for DNA damage, DNA repair, and mutation. The organization of these perturbations across levels of biological response could be incorporated into an engineering “failure model” [20] or for use in a more quantitative process as in genetic progression and waiting-time models [25]. In either of these modeling approaches, the degree of perturbation and adversity would be measured by a *composite probability* incorporating the contributions from the sequential stages of increasing pathway activation obtained by dose-response characterization of the pathway assays. The overall goal in such an aggregate analysis is to estimate, with some degree of confidence, regions of exposure with no increase in the frequency of mutation, as a result of integrated control through DNA repair, cell cycle delay or arrest, and apoptosis.

In a very real sense, the point about evaluating regions of exposures that are expected to be without effect, i.e., to be safe, is a departure from the current practice of estimating risks from high-dose animal exposures. The wording of an exposure recommendation would be that a compound with a mutagenic, DNA–reactivity-related mode of action is likely to cause cancer with prolonged, higher-dose exposures in humans. However, there would not be a quantitative estimate of risk, as is now the standard practice after determining the incidence of cancer in animals exposed to high doses of the test compound.

### Dynamic pathways underlying biological response

**Figure 4:**
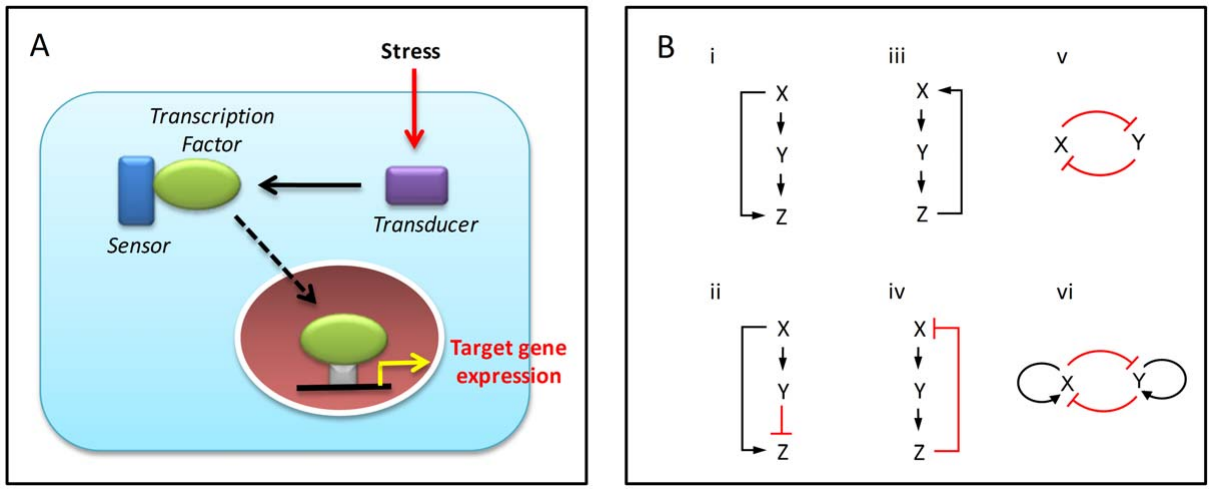
Stress response pathways and network motifs. (**A**) **Typical structure of a stress response pathway** (adapted from Simmons et al. [26]). The so-called eight canonical stress response pathways, conserved broadly across eukaryotes, have a common structure (common motifs) for sensing damage and mounting a transcriptional response to counteract the stress. (**B**) **Common network motifs in intracellular response pathways.** Three elements, (genes/proteins) X, Y, and Z, in a pathway can regulate each other to form: (**i**) a negative feedback loop; (**ii**) a positive feedback loop; (**iii**) a coherent feed-forward loop, where X activates Y, and both X and Y activate Z; and (**iv**) an incoherent feed-forward loop, where X activates both Y and Z, but Y suppresses Z. Two transcription factors X and Y can regulate each other through, for instance: (**v**) a double-negative feedback loop; or (**vi**) a double-negative feedback loop with positive autoregulation. Sharp arrows denote activation; flat arrows denote suppression.

The biological effects of a chemical or hazardous substance at the level of individual cells are mediated by cognate “receptor” molecules and downstream signaling and transcriptional networks (toxicity pathways). The subsequent changes in the state and dynamic behavior of these networks form the basis of the particular shape of the dose-response curve for specific phenotypic end points. There appear to be a finite, small number of core *stress-response pathways* that are activated by cells in response to various chemical stimuli to maintain homeostasis, or to make specific cell-fate decisions like apoptosis [26]. Examples include the oxidative stress response, heat-shock response, DNA-damage response, hypoxia, and endoplasmic reticulum stress pathways – all of which feature a common architecture consisting of a transcription factor, a “sensor,” and a “transducer” and are present in all cell types of an organism [26] (Figure 4A). Typically activated at concentrations of chemicals significantly lower than those that lead to adverse effects at the organism level, this suite of pathways can be assayed as a group to serve as predictors of potential cell damage [26], [27]. A second group of toxicity pathways are those related to activation of specific endogenous receptor pathways, such as estrogen, androgen, and thyroid hormone signaling.

These canonical stress-response pathways are in turn made up of a core set of functional *regulatory network motifs* that underlie cellular homeostasis, decision making, and phenotypic transitions. Discovered from detailed investigation of transcriptional regulatory networks in the bacterium *Escherichia coli* [28] and the budding yeast *Saccharomyces cerevisiae* [29], these motifs all have a characteristic structure and the capacity to perform specific information-processing functions [30], [31], [32] (Figure 4B). Examples include (a) *negative feedback*, which enables homeostasis and acceleration of response time in gene circuits [33], [34]; (b) *positive feedback*, which generates switching behavior between multiple phenotypic states [35]; (c) the *coherent feed-forward loop*, which can introduce a time delay in activation as well as detect persistence in the activating signal [36]; and (d) the *incoherent feed-forward loop*, which can function as a pulse generator and response accelerator [37], [38]. These motifs can also act in combination to generate more complex regulatory patterns in transcriptional networks [30]. Similar motifs have been identified in the cells of higher organisms – for example in the circuits that control gene expression in the pancreas and the liver [39] as well as the regulatory circuits of human embryonic [40] and hematopoietic [41], [42] stem cells. Understanding the organization and quantitative behavior of these circuits, which are likely to be key components underlying toxic response, should lead to improved prediction of the cellular outcome of specific perturbations introduced by various chemicals.

The advent of the “-*omics*” era has enabled detailed characterization of the molecular signatures associated with particular perturbed or disease states. However, a mechanistic understanding of the underlying biological processes will require *more- focused quantitative analysis* of specific pathways and network motifs derived from these large-scale molecular signatures [43]. In particular, understanding the dynamic behavior of toxicity pathways will require stimulation of these pathways at a number of time points and at various concentrations of the activating chemical, rather than static snapshots of the molecular state [44]. Computational systems biology pathway (CSBP) models are expected to play a key role in this process, allowing mechanistic prediction of the dose-response based on pathway dynamics. Crucial to the intermediate objective of estimating *in vitro* adversity, CSBP models need to include the molecular circuits responsible for the basal operation of toxicity pathways in the absence of an external chemical stressor. The basal dynamics set up the background state from which additional perturbations will occur as the stress level increases. Quantitative characterization of the network circuits (from in vitro assays) will dictate the changes in response from basal state to activation. A properly implemented CSBP model would take such changes into account to predict the range of concentrations of stressors that would not produce appreciable adversity. Another advantage to development of these CSBP models would be the ability to assess pathway components that display polymorphisms in the human population to help identify sensitive subpopulations.

### Computational systems biology of DNA-damage response networks

**Figure 5:**
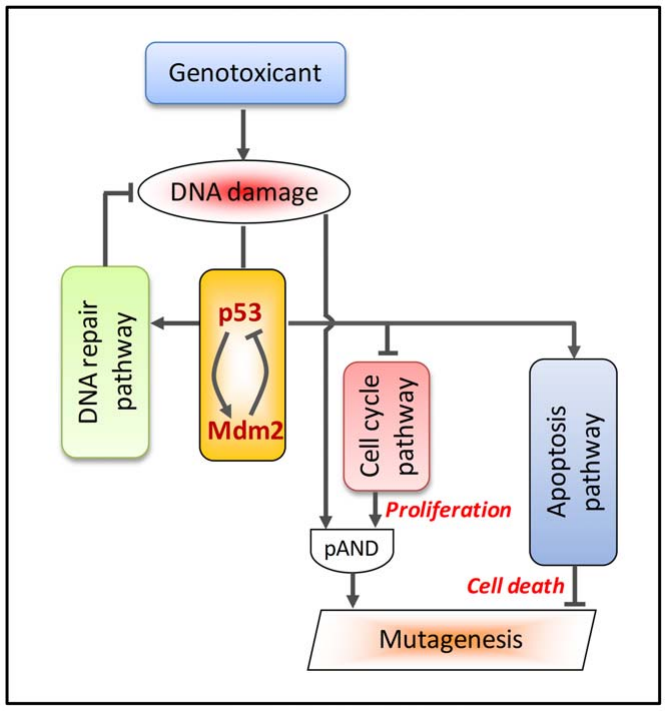
p53-mediated perturbation of DNA damage response pathways that affect mutagenesis. A negative feedback motif composed of p53, Mdm2, and others (not shown) can produce undamped oscillations in response to radiation-induced double strand breaks. A partial AND (pAND) gate is used to indicate that to produce inheritable mutations, both DNA damage and cell proliferation (DNA replication) are required, and that the two processes likely contribute to mutagenesis in a multiplicative manner. Activation of the apoptosis pathway works to mitigate mutagenesis by killing cells with severe or unrepairable DNA damage. Pointed arrows indicate activation and blunted arrows indicate inhibition.

In recent years there has been a great deal of progress in computational modeling of cellular response networks to predict their dose-response behavior (see http://www.thehamner.org/education-and-training/drm_workshop.html). In mammalian cells, the tumor suppressor protein p53 functions as an essential guardian of genomic stability – mutations in p53 are associated with nearly half of all cancers. DNA damage activates p53 through sensor molecules such as ataxia telangiectasia mutated (ATM)., Activated p53 in turn transcriptionally regulates several key pathways that cooperatively limit the increase in mutation frequency in affected cells (Figure 5) [45]. It is likely that p53 upregulates a number of DNA repair enzymes and itself acts as a component of the repair complex to enhance removal of damaged DNA products [46]. By transcriptionally activating p21 and other regulatory proteins, p53 also puts a brake on the cell cycle engine to arrest the cell at various mitotic stages [47]. What seems also important is that before the relatively slow p53-dependent transcriptional regulation is fully launched, several p53-independent post-translational mechanisms can be quickly activated to immediately delay/arrest the cell cycle [48], [49], [50]. The resulting slowdown or outright blockade of cell proliferation not only allows more time for DNA repair but also reduces the probability of mutation, because DNA replication is almost always required for DNA damage to become a heritable mutation. Therefore even though the tendency to mutate increases as a result of increased DNA damage, if cell proliferation rate is also reduced accordingly, there may not be any tangible increase in overall mutation frequency. If the degree of DNA damage in the cell is too severe to be effectively repaired, strongly activated p53 triggers the apoptosis pathway to eliminate the cell, which could otherwise mutate at a potentially dangerous high frequency [51].

It thus appears that perturbed cells use an incoherent feed-forward mechanism to control mutagenesis (Figure 5): the cell cycle arrest and apoptosis pathways together form the inhibitory arm, while DNA damage serves as the stimulatory arm (see also Figure 4B iv). Depending on the degrees of nonlinearity of the two arms, an incoherent feed-forward motif may generate either perfect adaptation or a non-monotonic response [52], [53], which can lead respectively to threshold behavior or to hormetic dose-response for mutagenesis/carcinogenesis [54]. Therefore, to understand the changes in mutation frequency in cells exposed to a particular concentration of genotoxic chemical, it is necessary to examine, in addition to DNA damage, the degree of activation of the pathways underlying DNA repair, cell cycle arrest, and apoptosis.

Understanding the time-dependent alterations in mutation frequency after exposure to genotoxic agents requires knowledge of the dynamics of p53 pathway activation. Recent studies have revealed rich dynamic behaviors in this pathway [55]; besides driving a number of the pathways described above, activated p53 can upregulate murine double minute 2 (Mdm2), a regulatory protein that promotes the degradation of p53 through the ubiquitin-proteasome pathway [56]. This negative feedback motif, together with other feedback regulations involving wild-type p53-induced phosphatase (WIP1), ATM, and checkpoint kinase 2 (CHK2) [57], generates oscillatory behavior of p53 and Mdm2 in response to DNA double-strand breaks induced by γ-irradiation [58], [59]. Interestingly, despite variations in pulse amplitude and period, the undamped oscillation of p53 activity in individual cells appears to be digital: (a) pulse amplitude and period are generally independent of the severity of DNA damage; and (b) cells either undergo uninterrupted oscillations at a characteristic frequency or do not oscillate at all, with the fraction of oscillating cells increasing with the dose of irradiation [58]. It remains to be seen, however, whether p53 oscillation is a conserved dynamic pattern in response to various genotoxic chemicals.

At this time the biological function of p53 oscillations is unclear. It is possible that multiple p53 pulses serve to measure the time elapsed subsequent to the onset of DNA damage. In that case, prolonged oscillation could act as a signal for persistent damage, triggering activation of the apoptotic pathway. Rigorous quantitative studies of p53 dynamics in the context of differential activation of DNA repair, cell cycle arrest, and apoptosis pathways are urgently required. To determine the low–dose- response behavior for mutation and the point of departure to adversity, it is also essential to understand how the p53 circuit behaves at basal conditions with only background DNA damage, and how its behavior is altered with increasing DNA damage under exposure to genotoxic chemicals. A recent study has suggested that at basal conditions p53 oscillates infrequently in a manner that is loosely coupled to the cell cycle without causing unnecessary cell cycle arrest [60]. A computational systems biology modeling approach that incorporates the oscillatory p53 and Mdm2 circuit in association with perturbations to downstream pathways for DNA repair, cell cycle, and apoptosis may allow a quantitative, mechanistic examination of regions of adequate repair function and excessive perturbations that result in enhanced frequency of mutation.

## Analysis

### Establishing regulatory standards

The combination of carefully designed *in vitro* assays and CSBP models of the structure and function of toxicity pathways can provide an estimate of the concentrations of chemicals *in vitro* that likely lead to excessive perturbation of the pathways. This concentration would comprise a redefined point of departure for risk assessment (Figure 2B). The process of setting acceptable exposure levels requires the ability to estimate human exposures that would give rise to tissue or blood (or plasma) concentrations equal to those concentrations found to be ‘adverse’ in *in vitro* assays with human cells. Such calculations would require contributions from the discipline of pharmacokinetic modeling, and more specifically, physiologically based pharmacokinetic modeling. In particular, a reverse dosimetry approach [61], [62] can be used to estimate the ranges of exposure expected to give rise to specific plasma or tissue concentrations. The overall process of moving from perturbations of a suite of toxicity pathways to a health risk assessment will require three different components: (a) appropriately designed *in vitro* toxicity pathway assays, (2) CSBP (computational systems biology pathway) models for extrapolation to lower *in vitro* concentrations, and (3) pharmacokinetic tools to convert active concentrations *in vitro* to expected human exposures that would yield equivalent tissue concentrations in exposed individuals.

### Implementing TT21C Toxicity Pathway-based Approaches

Three of the four key issues described earlier in this contribution in association with the NRC report: defining adversity, setting regulatory standards, and predicting toxicity, have been addressed above with the example of DNA-damage response. Adversity would be defined in relation to degrees of perturbation above basal stress/functional levels, while regulatory standards would be based on the active *in vitro* concentration and extrapolations with CSBP and pharmacokinetic model structures. The new approach will not be intended to predict risk in the particular quantitative fashion as is currently done. Today, based on high-dose responses, we estimate a dose expected to be associated with some degree of risk, e.g., 1 per 1,000,000. In contrast, the pathway approach would predict *safe regions of exposures*, e.g., those where perturbations will be insufficient to cause adverse outcomes at the cellular or organism level.

**Figure 6:**
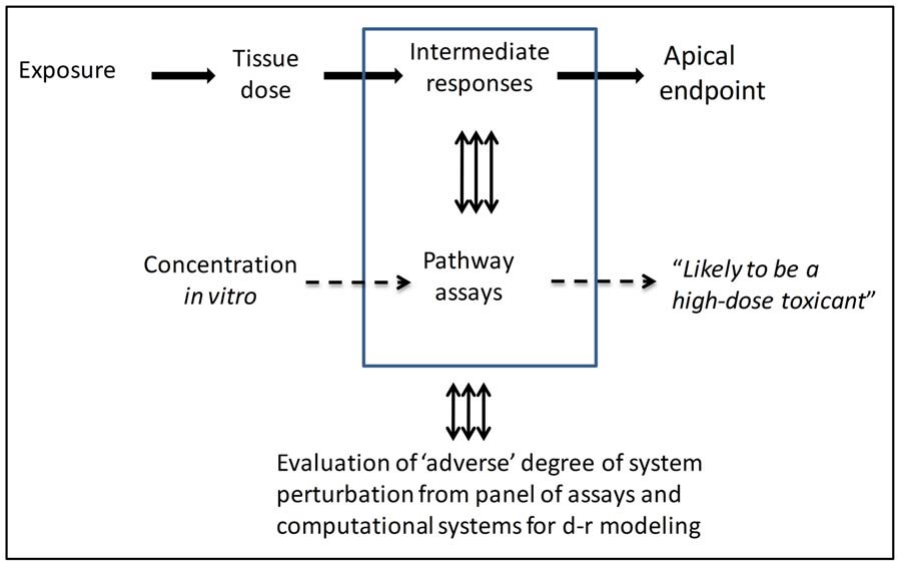
Using prototype pathways and compounds: the fast track for implementing the 2007 NRC TT21C vision. The next step in moving forward will be to see the entire 2007 TT21C vision placed in practice with a group of pathways and specific prototype compounds preferentially affecting the target pathways. The use of compounds with robust animal toxicity profiles will allow a mapping of *in vivo* responses associated with apical end points and intermediate end points against new *in vitro* toxicity pathway assay results. The comparison will permit a better sense of the relationship between *in vitro* and *in vivo* responses in key pathways and the relevant estimates of risk. Prototype compounds for the DNA-damage pathway could include DNA-damaging compounds – polyaromatic hydrocarbons, formaldehyde, and alkylating agents, etc. – and compounds causing DNA damage indirectly through oxidative stress pathways, such as flavonoids. The output of the two approaches in relation to risk estimates and estimates of safe region of exposure could also be compared to assess correspondence or lack of correspondence in the approaches.

We are left with the last of the four issues raised earlier: how to make change of this magnitude occur in the face of a long tradition of *in vivo* testing for assessing the hazards of chemicals for humans. These new approaches, based on mode of action, human biology, and a better appreciation of biological responses to stressors and pathway perturbation, promise improved throughput and reduced uncertainty in estimating the concentrations that cause various degrees of perturbation. Nonetheless, they will naturally be compared, in one way or another, to current approaches such as existing cancer bioassays to determine whether the new methods provide adequate safety for exposed human populations. The comparison of past practice and these more mode–of-action-based approaches will be difficult, but, in our opinion, can be made by using well-studied prototype compounds whose toxicity has already been examined with *in vivo* and *in vitro* assays (Figure 6).

These prototype compounds can support case studies for proof-of-concept exercises to evaluate mechanistic, toxicity–pathway-based approaches to the assessment of risks to human health. The choice of the prototype chemicals should be dictated by the specific stress pathways under consideration, e.g., DNA damage, endoplasmic-reticulum stress, and oxidative stress [26]. Alternatively, the chosen compound could have specific receptor-mediated targets – such as the estrogen receptor, the aryl hydrocarbon receptor, or the peroxisomal proliferator activating receptor (PPARs). These case studies could be more broadly useful in assessing how mode-of-action results should be used to inform risk assessment. Another value of these prototypes/case studies will be demonstration of the new TT21C paradigm in practice – showing how data would be collected, organized, and interpreted to make decisions about regions of exposure not expected to be associated with appreciable risk in human populations. Our laboratories are now actively pursuing various prototype studies examining DNA damage, oxidative stress, and PPAR-α signaling.

### Conclusions

A recent special issue of the *Journal of Toxicology and Environmental Health* contains reprints of the entire NRC report [63] and the EPA strategic plan proposed in response [64], as well as 14 articles discussing specific scientific tools that will become the foundation of the methods outlined in the TT21C vision [65], [66], [67], [68], [69], [70], [71], [72], [73], [74], [75], [76], [77], [78]. The tools discussed include computational systems biology (CSB) methods for pathway analysis [78] and “virtual tissue” models for predicting toxicity [76]. The NRC report has continued to generate interest globally. In their response [21] to the nine commentaries in *Toxicological Sciences* previously discussed, two members of the National Academy of Sciences committee noted that at the time of writing in early 2010, there had been some 75 presentations worldwide on the NRC report and the TT21C vision (these presentations were listed in supplementary materials of their article). In many ways, this proposed transformation of toxicity testing from the NRC report appears more and more to be an idea whose time has come. The transformation towards an *in vitro*, toxicity pathway test approach is especially timely and necessary in terms of the Seventh Amendment to the Cosmetics Directive in the EU and the looming reauthorization of the Toxic Substances Control Act in the US and the REACH program in the EU. REACH (Registration, Evaluation, Authorization and Restriction of Chemical substances) is a new EU regulation on chemicals and their safe use (EC 1907/2006) that went into force on June 1, 2007. Without a redirection of toxicity testing, these broad initiatives will require a large amount of conventional high-dose toxicity testing of questionable relevance for ensuring human safety in the use of chemicals. The TT21C paradigm provides a blueprint for change. Specific examples, such as the case study with the DNA–damage-repair pathway outlined here, will be instrumental in guiding these changes in designing new methods for testing toxicity and the tools such as CSBP and pharmacokinetic models necessary for interpretation of test results for assessing human safety.
